# Proteins in pregnant swine serum promote the African swine fever virus replication: an iTRAQ-based quantitative proteomic analysis

**DOI:** 10.1186/s12985-023-02004-3

**Published:** 2023-03-28

**Authors:** Jinke Yang, Xingguo Yuan, Yu Hao, Xijuan Shi, Xing Yang, Wenqian Yan, Lingling Chen, Dajun Zhang, Chaochao Shen, Dan Li, Zixiang Zhu, Xiangtao Liu, Haixue Zheng, Keshan Zhang

**Affiliations:** State Key Laboratory for Animal Disease Control and Prevention, College of Veterinary Medicine, Lanzhou University, Lanzhou Veterinary Research Institute, Chinese Academy of Agricultural Sciences, Lanzhou, 730000 China

**Keywords:** African swine fever virus, Pregnant swine serum, iTRAQ, DEPs, *PCNA*

## Abstract

African swine fever (ASF) is a severe infectious disease caused by the African swine fever virus (ASFV), seriously endangering the global pig industry. ASFV possesses a large genome, strong mutation ability, and complex immune escape mechanisms. Since the first case of ASF was reported in China in August 2018, it has had a significant impact on social economy and food safety. In the present study, pregnant swine serum (PSS) was found to promote viral replication; differentially expressed proteins (DEPs) in PSS were screened and identified using the isobaric tags for relative and absolute quantitation technology and compared with those in non-pregnant swine serum (NPSS). The DEPs were analyzed using Gene Ontology functional annotation, Kyoto Protocol Encyclopedia of Genes and Genome pathway enrichment, and protein–protein interaction networks. In addition, the DEPs were validated via western blot and RT-qPCR experiments. And the 342 of DEPs were identified in bone marrow-derived macrophages cultured with PSS compared with the NPSS. The 256 were upregulated and 86 of DEPs were downregulated. The primary biological functions of these DEPs involved signaling pathways that regulate cellular immune responses, growth cycles, and metabolism-related pathways. An overexpression experiment showed that the *PCNA* could promote ASFV replication whereas *MASP1* and *BST2* could inhibit it. These results further indicated that some protein molecules in PSS were involved in the regulation of ASFV replication. In the present study, the role of PSS in ASFV replication was analyzed using proteomics, and the study will be provided a basis for future detailed research on the pathogenic mechanism and host interactions of ASFV as well as new insights for the development of small-molecule compounds to inhibit ASFV.

**Supplementary Information:**

The online version contains supplementary material available at 10.1186/s12985-023-02004-3.

## Introduction

African swine fever (ASF) is an acute, febrile, and highly contagious disease of domestic pigs caused by the African swine fever virus (ASFV), with an acute infection mortality as high as 100% [1–3]. Currently, there are no widely recognized effective vaccines for ASF, and disease control relies on limited biological control measures such as rapid diagnosis, animal culling, and regional control. The ASF has caused serious losses to animal husbandry and global food security [4]. Pigs infected with the disease typically show clinical symptoms such as generalized hemorrhage, respiratory disorders, and neurological symptoms, characterized by a short disease duration and high mortality [5–7]. ASF was first reported in Kenya in 1921; in the following century, it has gradually spread to Europe, including Lithuania, Estonia, Poland, and Latvia [8–10]. In August 2018, an ASF epidemic was first reported in Liaoning Province, followed by successive reports of ASF epidemics in Vietnam and other countries. The rapid, continuous spread of ASFV through European and Asian countries poses a significant threat to pig breeding and human quality of life [11–14].

ASFV belongs to the *Asfarviridae* family and is the only known insect-borne DNA virus. Its genome size is approximately 170–190 kb, depending on the virus strain. The ASFV genome is a linear, double-stranded, closed DNA molecule with an inverted terminal and repeat hairpin loop and consists a conserved region of approximately 125 kb in the middle and a variable region encoding multigene families at both ends [15, 16]; the virus particles are enclosed in an icosahedral capsule. ASFV replication primarily occurs in the cytoplasm; however, the nucleus can also be the site of viral DNA synthesis during the early stages of infection [17]. The virus can affect wild and domestic pigs of almost all breeds and ages. The incidence of ASFV infection in large-scale farms showed a sex trend, with the infection in sows occurring earlier than in boars. Moreover, compared with other pig herds, pregnant sows showed the earliest onset and experienced more serious symptoms after infection. In some pregnant sows, the disease onset led to abortion and stillbirth, and the mortality reached 100% after 7 days [18]. Studies have revealed the presence of high estrogen levels in pregnant sows until the end of the gestation period [19]. Therefore, estrogen may be an important factor influencing ASFV replication. However, pregnant swine serum (PSS) contains complex components and multiple proteins, and the effect of PSS on ASFV infection and pathogenesis remains unclear.

In recent years, several studies have found that females often have stronger innate and adaptive immune responses compared with males, which may be attributed to genetic and physiological differences between sexes, thereby resulting in different immune responses to the virus [20–26]. Hepatitis B virus (HBV) is one of the most common human pathogens worldwide. The incidence of HBV-related hepatocellular carcinoma in males and females was approximately 6:1 [27]. The high estrogen levels, which are typical during pregnancy, might be related to the immune response to viral infection in females. In the early stage of human immunodeficiency virus type 1 (HIV-1) infection, compared with men, viremia was significantly lower and CD4^+^ T cell losses were twice as high in women. These differences were related to female β-estradiol (E2) levels, which contributes to the activation of CD4^+^ T cells and inhibition of viral replication [28]. In addition, some studies have reported that in mice infected with the influenza virus, E2 treatment resulted in lower morbidity and mortality, indicating that E2 exerts antiviral effects. In addition, multiple studies have shown that progesterone (P4) could be involved in the regulation of viral replication. P4 treatment resulted in a prolonged disease course, earlier disease onset, and an increase in overall morbidity and mortality in mice [29]. However, injecting P4 into adult female mice without P4 protected them from influenza A virus pneumonia and increased their survival [30]. At present, research on estrogen participation in viral infection chiefly focuses on hepatitis and influenza viruses; it was also reported for SARS-CoV-2 [31]. Besides high estrogen levels, serum from pregnant individuals contains numerous other complex components. Cytokines, such as *TNF-α*, *IL-1β*, and *IL-6*, were upregulated in the serum of pregnant women infected with the H1N1pdm 2009 influenza virus, indicating the complex role played by serum from pregnant individuals in the regulation of viral replication.

The development of proteomics and in particular, the isobaric tags for relative and absolute quantitation (iTRAQ) technology, has facilitated a better study of the interaction of viruses and host cells, thereby helping to investigate the pathogenesis of various diseases [32–35]. Despite the research on the role of estrogen in the regulation of viral replication, there is limited information on whether pregnant serum is involved in the proteomics of ASFV-infected host cells. In the present study, we observed the clinical symptoms of pigs with ASF and found that pregnant sows experienced the earliest disease onset, which was highly serious and led to abortion. PSS and non-pregnant swine serum (NPSS) were collected and added to bone marrow-derived macrophages (BMDMs). The differentially expressed proteins (DEPs) produced by the addition of PSS and NPSS were analyzed and identified using iTRAQ technology; specific DEPs were observed to affect ASFV replication, suggesting that PSS is involved in the regulation of ASFV replication. The present study provides a novel basis for the detailed research of the pathogenic mechanism and host interactions of ASFV as well as a new insight toward potential antiviral targets.

## Materials and methods

### Cell culture

Microbiological associates-104 (MA104) cells were cultured in Dulbecco’s modified Eagle medium (Thermo Scientific, USA) with 10% heat-inactivated fetal bovine serum (FBS, Thermo Scientific) [36]. BMDMs were obtained from the bone marrow of leg bones of pigs aged 30–40 days old and induced to differentiate into mature BMDMs in RPMI 1640 medium (Thermo Scientific) containing 20% FBS and porcine colony stimulating factor [37]. All cells were cultured in 5% CO_2_ in air at 37 °C. The pigs were examined to determine the absence of contamination with pathogens such as bacteria, fungi, classical swine fever virus, porcine reproductive and respiratory syndrome virus, pseudorabies virus, and porcine circovirus type 2.

At 24 h after exogenous plasmid transfection, the MA104 cell culture medium was changed to serum-free medium, ASFV (GS/CN/2018, MOI = 1) was inoculated and incubated for 2 h, and the medium was switched to fresh serum-containing medium (5% FBS). Treated cell samples were collected at 12, 24, 36, and 48 h post ASFV infection.

All experiments involving ASFV were performed in the Animal Biosafety Level 3 facilities at the ASFV Regional Laboratory (Lanzhou) and were performed according to the Assessment and Accreditation of Laboratory Animal Care International and the Institutional Animal Care and Use Committee guidelines (license no. SYXK [GAN] 2014-003).

### Antibodies and reagents

The mouse monoclonal antibody proliferating cell nuclear antigen (*PCNA*; 2586 T) was purchased from Gansu Yuzekang Trading Co., Ltd. (Lanzhou, China). The mouse and rabbit monoclonal antibodies *MASP1* (M053577S) and *JUNB* (T55894S), respectively, were purchased from Abmart Pharmaceutical Technology Co., Ltd. (Shanghai, China). The mouse monoclonal antibodies against Myc (2276S) and *β-actin* (3700S) were purchased from Shanghai Youyongwei Biotechnology Co., Ltd. (Shanghai, China). The mouse monoclonal antibody bone marrow stromal cell antigen 2 (*BST2*; 66919-1-Ig), HRP goat anti-rabbit IgG (H + L) (SA00001-2), and HRP goat anti-mouse IgG (H + L) (SA00001-1) were purchased from Lanzhou Lihe Biotechnology Co., Ltd. (Lanzhou, China). The mouse monoclonal antibodies *P72* (*B646L*) and *P30* (*CP204L*) were provided by the Foot and Mouth Disease Emerging Incidence and Epidemiology Team.

### Plasmids and transfections

Eukaryotic expression plasmids were constructed to insert *PCNA* (gene ID: 692192), *MASP1* (gene ID: 100152125), and *BST2* (gene ID: 100302088) into the vector pCDNA3.1(+)-Myc-C via gene cloning (Wuhan Gene Create Biological Engineering Co., Ltd., China). To transfect the relevant plasmids, MA104 cells were seeded onto the indicated plates at the appropriate density and grown to 80% confluence according to the experimental protocol. The constructed plasmids were transfected using jetPRIME® transfection reagent (Polyplus-transfection SA, France).

### Preparation, extraction, and enzymatic hydrolysis of protein samples

Both PSS and NPSS were provided by the Foot and Mouth Disease Emerging Incidence and Epidemiology Team. BMDM cells were recovered and cultured in RPMI 1640 medium with 10% PSS or NPSS for use in two replicate experiments; samples were collected and processed 24 h later. Proteins were extracted via SDS lysis (4% SDS, 1 mM DTT, 150 mM Tris–HCl, 1% protease inhibitors) and quantified using BCA [38]. Then, an appropriate amount of protein from each sample was subjected to trypsin hydrolysis via filtered proteome preparation (FASP); the peptides were desalted using the C18 cartridge and quantified (OD_280_) [38].

### iTRAQ labeling and strong cation exchange chromatographic fractionation

Peptides (100 μg) from each sample were labeled according to iTRAQ labeling kit (AB Sciex, Singapore). Each set of labeled peptides was mixed and graded using the AKTA Purifier 100 system. The absorbance at 214 nm was monitored during elution and the resulting solution was lyophilized and desalted using the C18 cartridge.

### LC–MS/MS data acquisition, protein identification, and quantitative analysis

The fractionated samples were separated using a nanoliter flow rate HPLC liquid phase system (EasynLC). The buffer solutions used were as follows: Solution A was 0.1% formic acid in water and Solution B was 0.1% formic acid in 84% acetonitrile in water. Following separation, mass spectrometric analysis was performed using the Q-Exactive mass spectrometer (Thermo Fisher, USA), and the molecular masses of polypeptides and polypeptide fragments were estimated using charge ratio. The mass spectrometry results were retrieved using Proteome Discoverer 1.4, following which protein identification and quantitative analysis were performed. Protein identification of these DEPs was performed by Shanghai Houji Biotechnology Co., Ltd. (Shanghai, China).

### Gene Ontology (GO) functional annotation and Kyoto Protocol Encyclopedia of Genes and Genome (KEGG) pathway enrichment analysis

GO (http://www.geneontology.org/) analysis and KEGG pathway enrichment analysis were performed on the identified DEPs to annotate the biological functions [39–42]. DEPs were screened for significant enrichment using the standard differential multiple of log 2 (|log2FoldChange| of > 1.2 with the *p* value of < 0.05).

### Protein–protein interaction (PPI) network construction and analysis

The detected DEPs were entered into the STRING software (https://tring-db.org) to construct a PPI interaction network diagram and perform network analysis [41].

### Real-time (RT)-qPCR

BMDM samples in serum-containing medium were processed for total RNA extraction. Total RNA in BMDMs was extracted using TRizol reagent (Thermo Fisher) and reverse transcribed using the PrimeScript RT kit (Takara, Japan). RT-qPCR was performed using the PowerUp™ SYBR™ Green Master Mix on the ABI StepOnePlus system. All data were analyzed using the StepOnePlus software, and the relative mRNA levels of these genes were normalized to porcine *GAPDH* mRNA levels [43]. In addition, the relative mRNA expression of DEPs was calculated using the comparative cycle threshold (2^−∆∆CT^) method. Primer sequences are presented in Table 1.

**Table 1:** Primers and oligonucleotides used in this study

Primers	Sequences (5′-3′)
MASP1-Forward	TAGAGGACCATCCTGAGGTATC
MASP1-Reverse	GATACTGTGGCTCTGGGTATTG
PCNA-Forward	CACTGAGGTACCTGAACTTCTTTA
PCNA-Reverse	CGATCTTGGGAGCCAAATAGT
VTN-Forward	CAGGAGGCTACAACTATGATGAC
VTN-Reverse	TTCACTCGGTAGTACTCCTCTC
JUNB-Forward	CAGGAGCGCATCAAAGTAGAG
JUNB-Reverse	CCTTGAGTGTCTTCACCTTGTC
SFXN1-Forward	ATGGCCATCCCTCCATTTATC
SFXN1-Reverse	GCACAAAGGTGTAGCAAATACC
PM20D1-Forward	ACACAGACAGCAGACACTATTC
PM20D1-Reverse	CGTAGGCTTGGACTGAGATTC
BST2-Forward	ATCTGCAGCACCAGCTAAC
BST2-Reverse	ACTTTGCGTCTTCTCCATCTC
SEC14L1-Forward	AGGAGCTGGAGAACGAAGA
SEC14L1-Reverse	GAGGCGTCCACAATCTGAAT
SERPINB2-Forward	GAAGAAACTGAATGGGCTTTATCC
SERPINB2-Reverse	CTGAGCCTTTAGGTCTGCTAAG
SLA-1-Forward	AGGAGTATTGGGATGAGGAGAC
SLA-1-Reverse	ATTCTCTGGAGGGTGTGAGA
GAPDH-Forward	CAATGACCCCTTCATTGACC
GAPDH-Reverse	ATCACCCCA TTTGAT GTTGG

Samples were collected at the designated timepoints after ASFV inoculation on MA104 cells. The copy number of ASFV genomic DNA was detected via RT–qPCR using the ASFV *P72* gene as a target. The sample DNA was extracted using QIAamp DNA Mini Kit (Qiagen, Germany) followed by RT-qPCR performed with the Bio-Rad system. The sequences used were as follows: ASFV-*P72*-R: 5′-CTGCTCATGGTATCAATCTTATCGA-3′; ASFV-*P72*-F: 5′-GATACCACAAGATCAGCCGT-3′; and TaqMan: 5′-CCACGGGAGGAATACCAACCCAGTG-3′. The ASFV genome copy number was calculated using the standard curve [22].

### SDS-PAGE and western blot

For western blotting, proteins were separated using SDS-PAGE (80 V, 30 min; 120 V, 60 min). Proteins were then transferred to nitrocellulose membranes (Pall, USA) (100 V, 90 min) in an ice bath, sealed with 5% skimmed milk, washed with TBST (containing 0.1% Tween 20), incubated overnight at 4 °C, and washed three times for 5 min using TBST. Then, they were incubated with the indicated secondary antibodies for 1 h at room temperature before washing using TBST. Finally, an electrochemiluminescence solution (Advansta, USA) was added to the incubator, and an image was obtained using an Odyssey infrared imaging system.

### Statistical analysis

All experimental data were presented as mean ± standard error values of three independent experiments. GraphPad Prismv.8 (San Diego, USA) was used to analyze the significance of the experimental results. The gray value analysis was completed using the ImageJ software (National Institutes of Health, USA). The *p* value of < 0.05 was considered significant, and *p* values of < 0.01 and < 0.001 denoted high significance.

## Results

### Promotion of ASFV replication by PSS

**Fig. 1:**
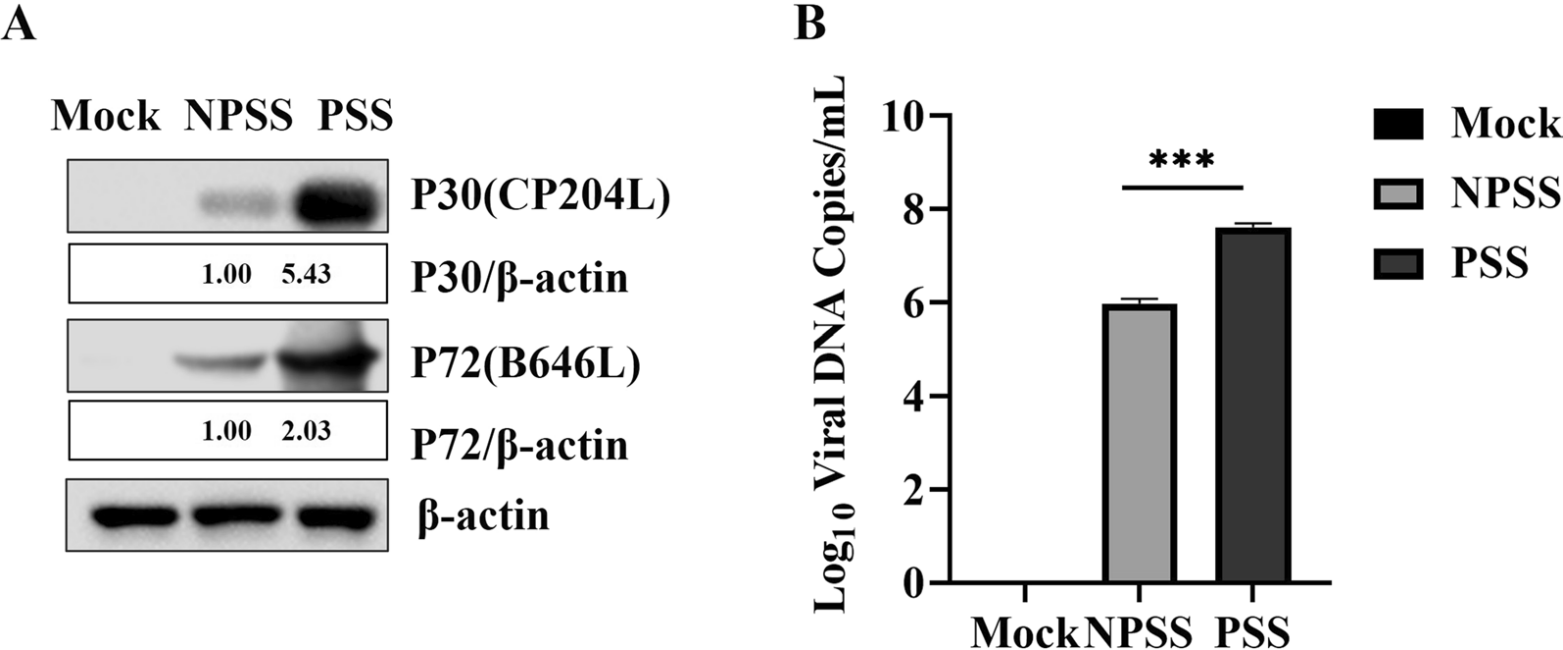
PSS promoted ASFV replication. **A** and **B** BMDMs were cultured in RPMI 1640 medium with 10% NPSS and 10% PSS for 24 h; then they were infected with ASFV (MOI = 0.5). Samples were collected 24 h after infection. The cell supernatant was collected in duplicate, and ASFV genomic copies were detected using absolute quantitative analysis after repeated freezing and thawing for three times. Another sample was used for western blot to detect protein expression of ASFV *P72* and *P30*, using *β -actin* as the control. The gray value analysis was performed using the ImageJ software. Experimental data are expressed as the mean ± standard deviation values of three independent experiments. *** indicating* p* values of < 0.001 was considered statistically significant

The BMDMs cultured in RPMI 1640 cell culture medium with 10% PSS and 10% NPSS and inoculated with ASFV (MOI = 0.5) were acquired at the designated timepoints. The expression levels of ASFV *P72* (*B646L*) and *P30* (*CP2043L*) in cells cultured with PSS were significantly higher those in cells cultured with NPSS; *β-actin* was used as reference. ASFV genome copies in the PSS group were higher than that in the NPSS group (Fig. 1A and B). These results indicated that PSS could promote ASFV replication.

### DEPs identified using iTRAQ LC–MS/MS

**Fig. 2:**
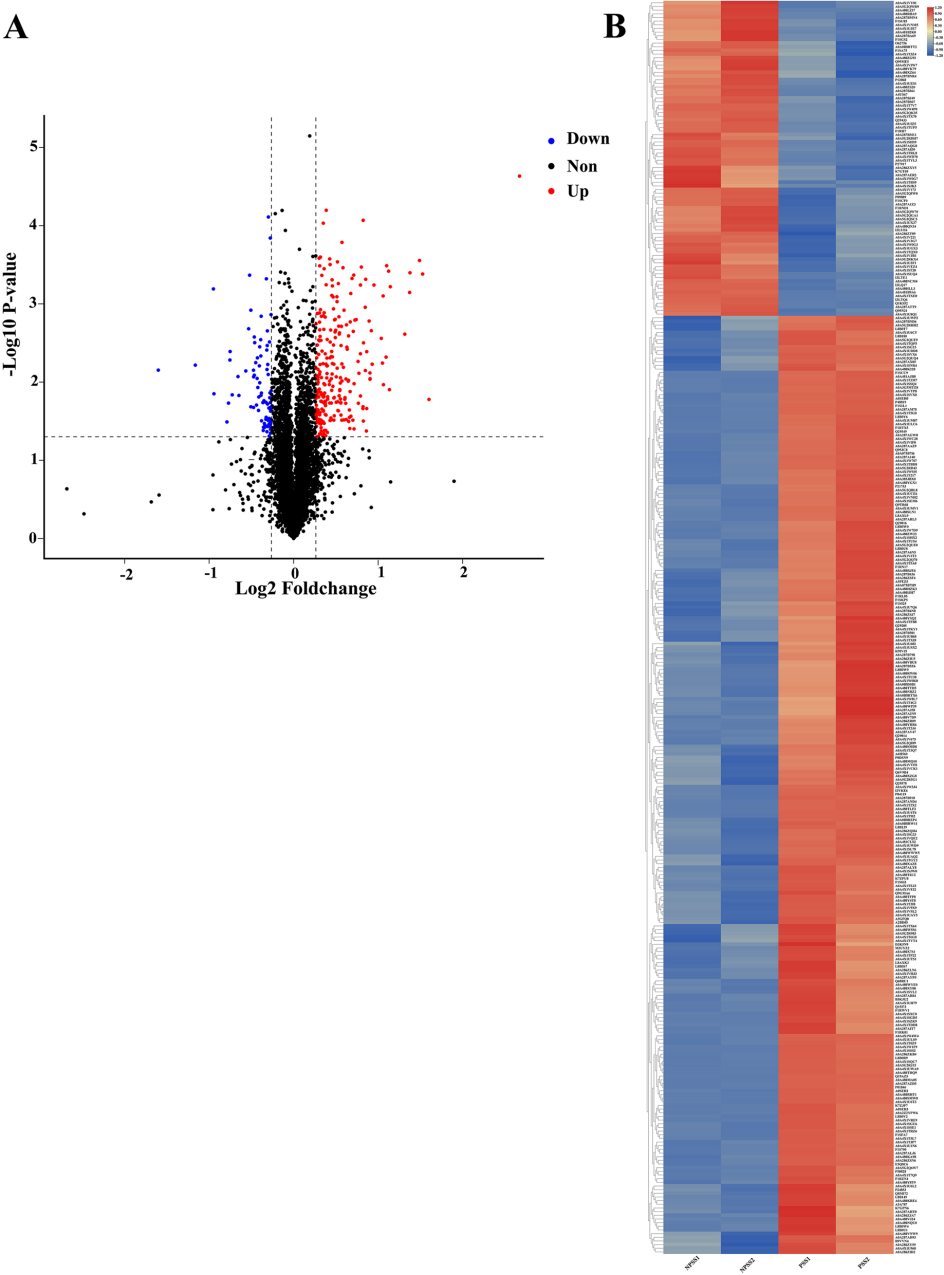
Volcano and cluster heat map analysis of DEPs between PSS and NPSS. **A** Red, blue, and black dots in volcano plots represent proteins that were upregulated, downregulated, or not significantly changed, respectively. The absolute *x*-axis value indicates the fold change of DEPs; the higher the absolute *x*-value (|log2FoldChange|), the greater the fold change. The *y*-axis indicates the significant *p* value (log transformation with base 10) of DEPs; larger *y*-values indicate smaller *p* values. **B** Hierarchical clustering results are presented as tree heatmaps. Each row in the figure represents a protein (the ordinate are proteins with significantly different expressions); each column represents a group of samples (the abscissa indicates the sample information). The logarithmic value (log2FoldChange) of significant differences in protein expression is presented in different colors in the heat map, where red and blue represents a relatively high and low protein content, respectively. In the figure, the NPSS groups are presented in the first two columns and the PSS groups are presented in the latter two columns

iTRAQ is a high-throughput screening technology commonly used in quantitative proteomics [44, 45]. iTRAQ technology has widely been used in the study of viral infection and disease development and for screening of biological markers [34, 46, 47]. To understand the role of PSS in ASFV replication, BMDMs were cultured with PSS and NPSS for 24 h, and DEPs in PSS and NPSS were identified using iTRAQ technology. Overall, 5830 proteins were identified; 342 DEPs were found in the PSS comped with the NPSS group (Fig. 2A). Of the 342 DEPs identified in the PSS group, a volcano diagram showed that 256 were upregulated and 86 were downregulated (Additional file 1: Table 1). The overall expression trend and distribution of all DEPs are shown using a clustering heat map (Fig. 2B). DEPs were screened based on |log2FoldChange| of > 1.2 and the *p* value of < 0.05. The results showed that the addition of PSS changed the expression profile of various intracellular proteins, indicating that DEPs in PSS may play a role in cellular response to ASFV infection.

### GO functional annotation and KEGG enrichment analysis of DEPs

**Fig. 3:**
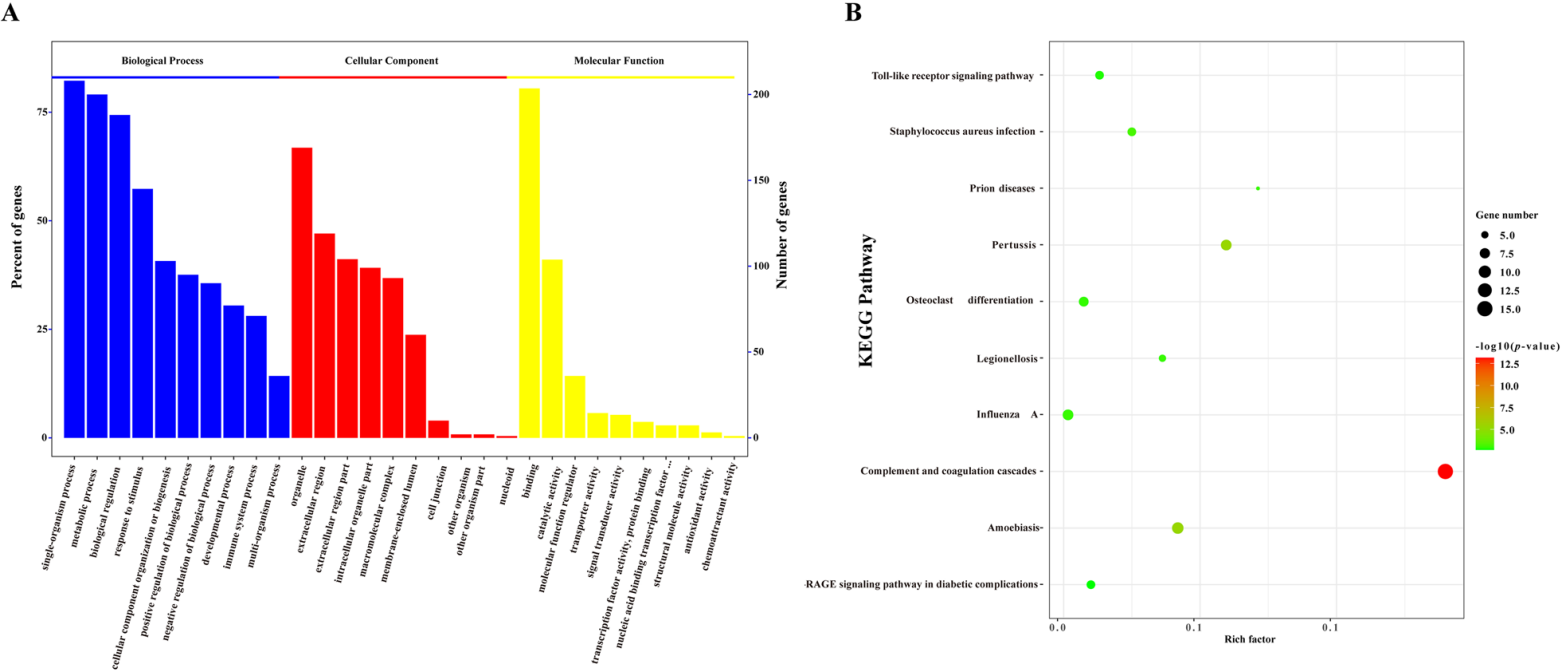
GO functional annotation and KEGG pathway enrichment analysis of DEPs between PSS and NPSS. **A** The abscissa represents enriched GO functional components. The top 10 items of significance for the enrichment analysis of Biological Process (BP), Cellular Component (CC), and Molecular Function (MF) are shown. The left ordinate indicates the number of proteins for each entry as a percentage of the total number of submitted proteins, and the right ordinate indicates the number of proteins for each entry. **B** The vertical axis indicates the top 10 significantly enriched KEGG pathways. The abscissa indicates the enrichment factor (rich factor ≤ 1) of each KEGG pathway. The bubble color indicates the significance of the enriched KEGG pathway. The color gradient (from green to red) indicates the *p* value, with red, indicating small *p* values corresponding to higher significance of the KEGG pathway enrichment. Bubble size indicates the number of DEPs involved in the KEGG pathway. Data were analyzed using the Omics Bean software, and protein enrichment analysis was plotted using the R software

To further reveal the potential biological functions of the DEPs in PSS, these DEPs were subjected to GO functional annotation and KEGG enrichment analysis. The GO analysis indicated that DEPs were primarily involved in biological processes such as developmental process, immune system process, biological regulation, and metabolic process. Moreover, the identified main molecular function of DEPs was to participate in the regulation of protein activities, such as protein binding and catalytic and hydrolytic activities (Fig. 3A). Moreover, studies have shown that serum components play a role in the regulation of disease occurrence and viral infection in the body [48–50]. To further analyze the biological function of DEPs, we used KEGG enrichment analysis. KEGG analysis revealed that most DEPs played a regulatory role in the complement and coagulation cascades, Toll-like receptor signaling pathway, AGE–RAGE signaling pathway, and other signaling pathways involved in the initiation of viral infection (Fig. 3B). Accordingly, DEPs in PSS may potentially regulate the immune response, cell metabolism, and antiviral signal transduction in the host, thereby influencing cellular responses to ASFV infection.

### PPI network analysis of DEPs

**Fig. 4:**
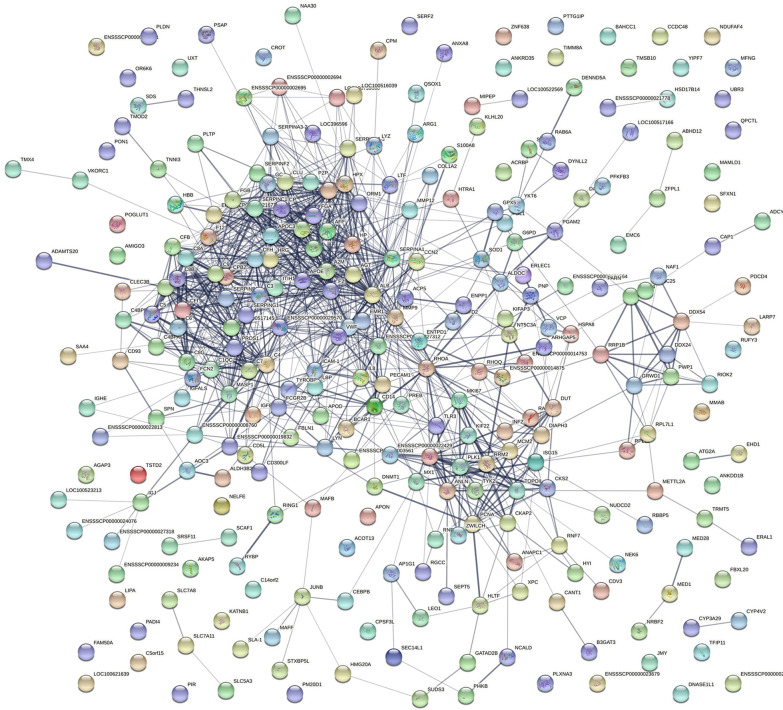
PPI network of DEPs between PSS and NPSS. The PPI network map was constructed using the STRING software. The nodes represent proteins and the lines represent PPIs. Yellow nodes in the network indicate DEPs, and blue nodes indicate other proteins in the interaction network database that could directly interact with DEPs. Proteins are indicated by their respective gene ID numbers

The interaction between these DEPs was further explored based on GO and KEGG analyses. A PPI network of DEPs was constructed using the STRING software to comprehensively analyze the interactions between the DEPs in PSS and host proteins. In the PPI network, the number of edges (1,043) was significantly higher than the number of nodes (237; Fig. 4), indicating that several DEPs do not directly intervene but bind to other host proteins to exert their biological functions in functional protein complexes, which are involved in ASFV replication. Therefore, the role of these DEPs in PSS warrants further investigation.

### Confirmation of DEPs

**Fig. 5:**
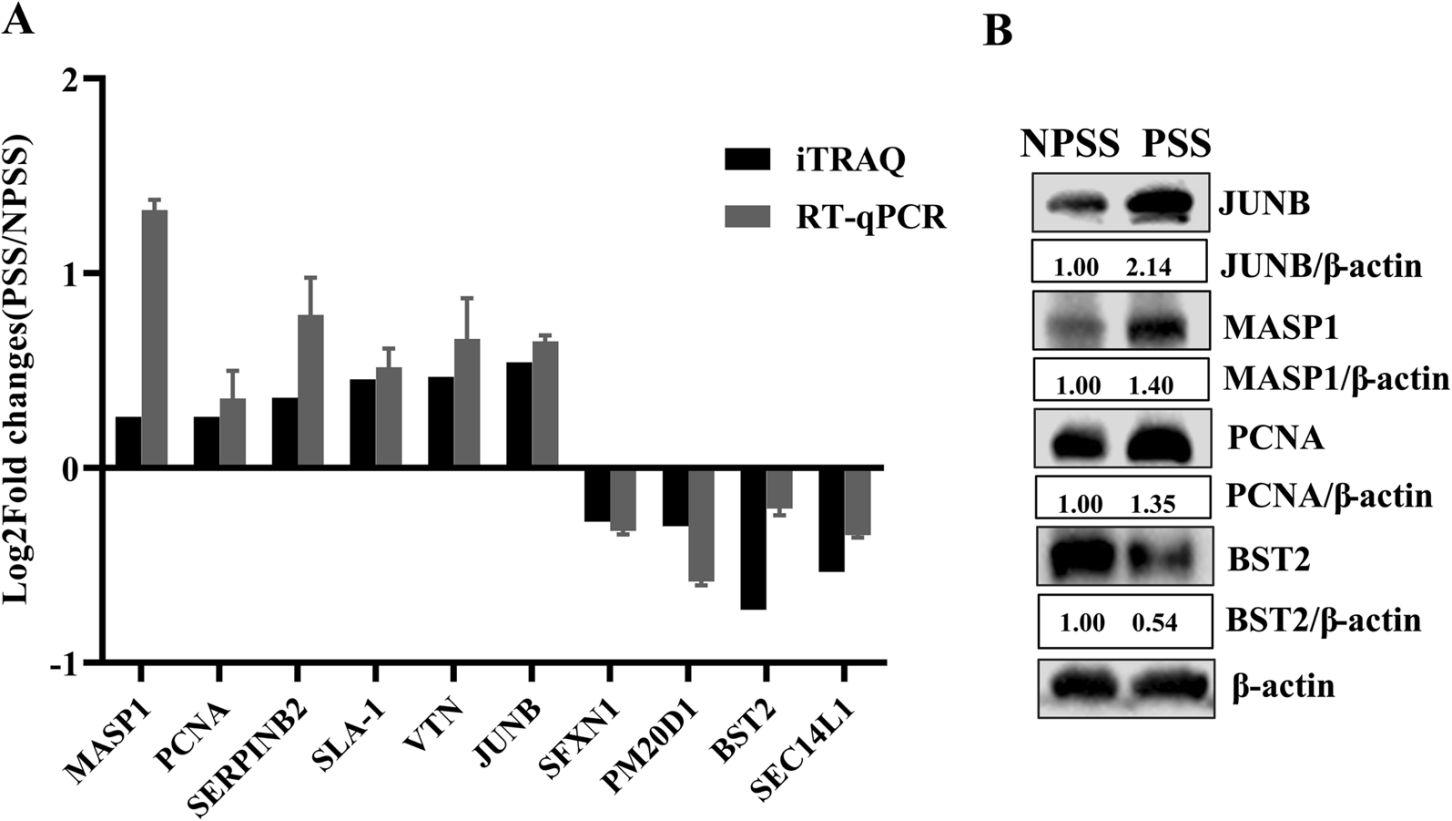
Validation of DEPs between PSS and NPSS based on iTRAQ technology. **A** The *MASP1*, *PCNA*, *SERPINB2*, *SLA-1*, *VTN*, *JUNB*, *SFXN1*, *PM20D1*, *BST2*, and *SEC14L1* were randomly selected from the upregulated or downregulated DEPs to verify changes in their mRNA transcript levels; *GAPDH* was used as an internal reference to determine iTRAQ feasibility using the 2^−∆∆CT^ normalization method. **B** iTRAQ accuracy was verified via western blot using the randomly selected upregulated proteins *PCNA*, *JUNB*, and *MASP1* and the downregulated protein *BST2*. The analysis of gray values in the figures was performed using the ImageJ software

A total of 10 differential proteins were randomly selected from PSS and NPSS, and their mRNA transcript levels were detected using RT-qPCR. *GAPDH* was used as the internal reference. The differential patterns of mRNA abundance of the genes in the DEPs were quite similar to the patterns of changes observed in the LC–MS/MS data (Fig. 5A). Four of these proteins were further randomly selected for protein level confirmation. As shown in Fig. 5B, *PCNA*, *JUNB*, and *MASP1* were significantly upregulated, and *BST2* was significantly downregulated. The results showed that DEPs in PSS and NPSS screened using iTRAQ technology were dependable and could serve as reference for subsequent studies.

### Regulation of ASFV replication by DEPs

**Fig. 6:**
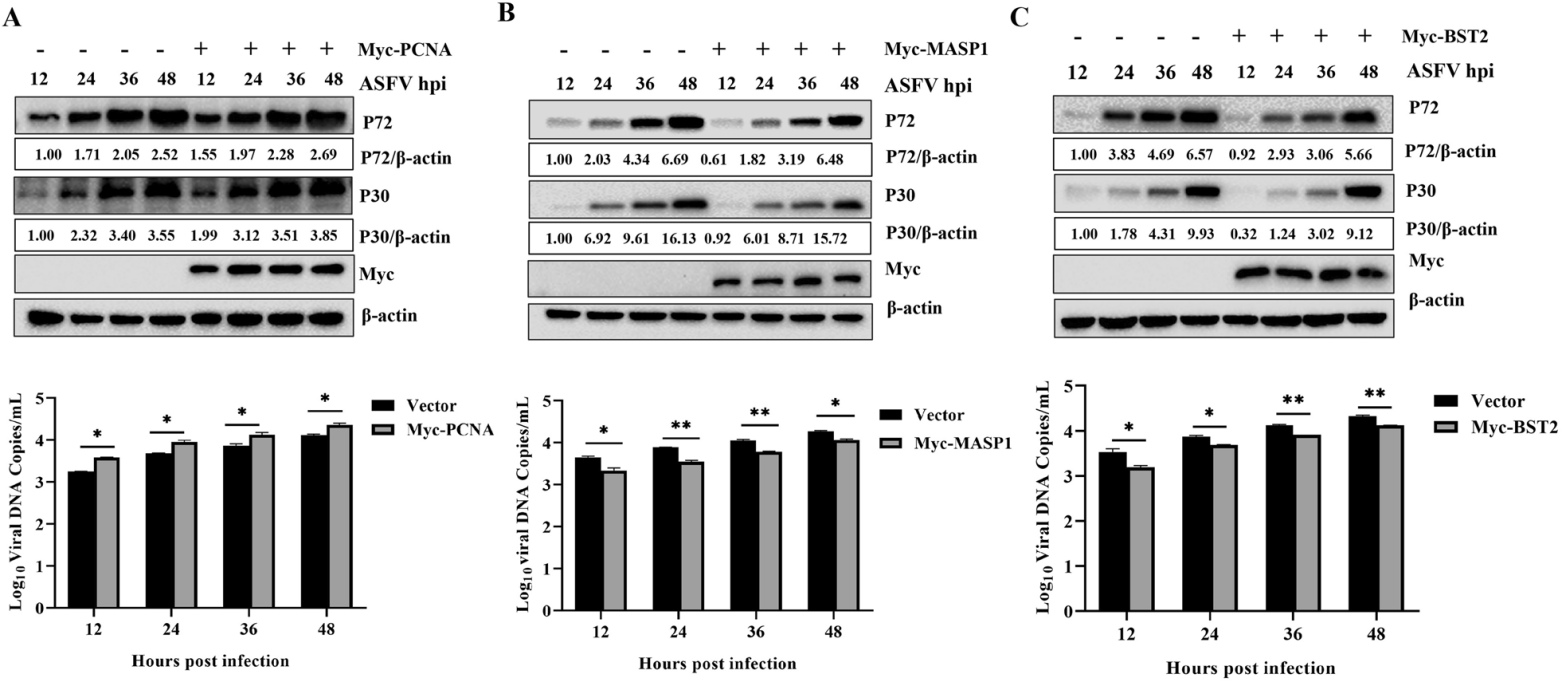
ASFV replication was regulated by DEPs between PSS and NPSS. MA104 cells were plated in 12 well plates at 80% confluency and transfected with the empty vector pCDNA3.1 (4 µg) or Myc-*PCNA* (4 µg), Myc-*MASP1* (4 µg), or Myc-*BST2* (4 µg). At 24 h after transfection, ASFV (MOI = 1) was added to the cells. Samples were collected at 12, 24, 36, and 48 h post infection. One sample was used to detect the genomic copies of overexpressed Myc-*PCNA* (**A**), Myc-*MASP1* (**B**), and Myc-*BST2* (**C**) at different ASFV infection timepoints in absolute quantitative experiments; the other sample was used to detect the effect of Myc-*PCNA*, Myc-*MASP1*, and Myc-*BST2* overexpression on ASFV *P72* and *P30* protein expression using western blot. The group transfected with the empty vector pCDNA3.1 was used as control. The gray value analysis was performed using the ImageJ software. The data are presented as mean ± standard deviation values of three independent experiments. * indicating *p* value of < 0.05 denoted statistical significance; ** indicating *p* value of < 0.01 denoted high statistical significance

To further identify DEPs involved in the regulation of ASFV replication in PSS, the upregulated proteins *PCNA* and *MASP1* and the downregulated protein *BST2* were selected for subsequent studies. *PCNA* is an accessory protein of DNA polymerase δ that can regulate DNA replication in eukaryotes by increasing polymerase processibility during lead strand extension [51, 52]. *MASP1* is an important protein involved in the mannose-binding lectin (MBL) pathway in the complement system and a key molecule that induces an immune response [53, 54]. *BST2* is a newly identified immune response factor capable of regulating replication of viruses, such as Kaposi’s tumor herpes virus (KSHV), porcine epidemic diarrhea virus, and Ebola virus [55–58]. To determine the effect of *PCNA*, *MASP1*, and *BST2* on ASFV replication, Myc-*PCNA*, Myc-*MASP1*, or Myc-*BST2* were transfected into MA104 cells, infected with ASFV (MOI = 1) at 24 h after transfection, and the processed samples were collected at 12, 24, 36, and 48 h post infection. The expression levels of the ASFV proteins *P72* and *P30* were significantly increased following *PCNA* overexpression, indicating that *PCNA* could promote viral replication in a time-dependent manner; moreover, ASFV copy number was consistent with the western blot findings, further indicating that *PCNA* could promote ASFV replication (Fig. 6A). ASFV replication was inhibited in cells overexpressing *MASP1* and *BST2*. The expression levels of the ASFV proteins *P72* and *P30* in these cells were lower than those in the empty vector control group cells, and there were fewer ASFV genome copies, indicating that *MASP1* and *BST2* can inhibit ASFV replication in a time-dependent manner (Fig. 6B, C). The above results indicated that specific DEPs in PSS play a role in the regulation of ASFV replication.

## Discussion

Since the first case of ASF reported in Kenya in 1921, it has rapidly spread to Europe and Asia and caused serious harm to food security and animal husbandry worldwide. The first case of ASFV was reported in China in 2018; since then, it has mainly been characterized as an endemic disease. Owing to the lack of commercial vaccines for protecting pigs, biological prevention and control measures have achieved little success. In addition, the continuous emergence of natural field-attenuated strains poses a challenge for the early diagnosis and control of ASF [59]. The clinical symptoms of swine infected with ASFV were typically first manifested in sows, with pregnant sows showing the earliest onset and severe symptoms compared with other swine groups; in pregnant sows, the disease led to abortion. This may be attributed to the high estrogen levels in pregnant sows, with some studies reporting that estrogen plays a regulatory role in viral replication; however, the influence of complex components in PSS on the infection and pathogenicity of ASFV remains unclear.

According to clinical findings and experimental validation, PSS played an important role in promoting ASFV (Fig. 1). This result was consistent with that result reported in the laboratory studies [60]. Steroid hormones reportedly are involved in immune regulation. Sex hormones are an important component of steroid hormone. For example, sex hormones could be involved in SARS-CoV-2 replication by affecting metabolic capacity and steroid hormones could inhibit the DNA synthesis of herpes simplex virus type 1, thereby inhibiting viral replication [61–63]. Moreover, estrogens have been involved in hepatitis E virus replication and play a regulatory role in HIV-1 transmission [64, 65]. With the development of bioinformatics, scientists have dedicated their efforts in revealing the regulatory role played by serum from pregnant individuals in virus-infected host cells. Exploring the detailed function and mechanism of serum from pregnant individuals in virus infection remains necessary.

In the present study, to screen the key factors affecting ASFV replication in PSS, DEPs in PSS and NPSS were screened using iTRAQ technology; we identified 342 DEPs in PSS (Fig. 2). GO functional annotation analysis of these DEPs showed that they are primarily involved in the growth metabolism and cycle regulation, protein modification, and immune response regulation of host cells (Fig. 3A). In the present study, DEPs with different functions were screened. *PCNA* is a key molecule in cell germinal development [66, 67]. *JUNB* is reportedly involved in the regulation of cell metabolic function. Moreover, *c-JUN* and *JUNB* transcription factors reportedly promote the proliferation of classical Hodgkin’s lymphoma tumor cells through the transport of G proteins [68]. The differential protein *BST2*, an antiviral natural immune protein, has a broad-spectrum inhibitory effect on envelope viruses, including retrovirus (HIV-1 and HIV-2), Ebola virus, KSHV, and rhabdovirus [69–73]. In the present study, KEGG enrichment analysis revealed highly significant changes in the complement and coagulation cascades, suggesting that the complement pathway plays an important role in regulating viral replication (Fig. 3B). Several viruses, such as poxviruses, SARS-CoV-2, and KSHV, achieve immune escape using targeted proteins in the complement system [74, 75]. In the present study, *MASP1*, as a key molecule of the MBL pathway in the complement system, could induce immune responses after activation [76]. In addition, the Toll-like receptor signaling pathway altered significantly, suggesting that the addition of PSS induces changes in the homeostasis of the immune response system. In this study, the *SEC14L* as a differential protein, which interacted with *RIG-I*, limited *RIG-I*-induced transcriptional activation of the *IFN-β* promoter, blocked the interaction of downstream effectors, and regulated viral replication [77]. The *ERAL1* was shown to promote *MAVS* polymerization downstream of the *RIG-I* signaling pathway, thereby actively regulating antiviral responses [78]. The above results suggested that DEPs play an important role in the host response to ASFV infection; however, the detailed functions and regulatory mechanisms of these DEPs require further investigation. Further analysis using the PPI network of DEPs showed that the number of edges was significantly higher than the number of nodes in the PPI network of the PSS and NPSS (Fig. 4). Based on the above analysis, PSS may play an important role in regulating organism homeostasis during ASFV infection. Moreover, the present study further validated the results of iTRAQ analysis for mRNA transcript and protein expression levels of selected DEPs, and these DEPs were found to be reliable and provided a basis for future research (Fig. 5).

Proteomic analysis suggested that DEPs, such as *MASP1*, *PCNA*, and *BST2*, play a key regulatory role in promoting ASFV replication in PSS. *PCNA* overexpression could promote ASFV replication, whereas *MASP1* and *BST2* overexpression could inhibit it (Fig. 6). *PCNA* is a cellular protein that acts as a DNA polymerase clamp during DNA replication, and N-Myc downstream regulatory gene 1 can directly interact with *PCNA*, forming a complex with *LANA* and *PCNA* to promote viral DNA replication [79]. Moreover, *PCNA* can target mouse autonomous parvovirus minute virus to degrade p21 through ubiquitination and promote viral replication [80]. In the present study, compared with NPSS, *PCNA* was upregulated in PSS, and *PCNA* overexpression could promote ASFV replication in its cytoplasm. Therefore, *PCNA* may be a cofactor in ASFV replication. *MASP1* is a key molecule that activates the lectin pathway in the complement system and is required for C3 convertase formation. *MASP1* can be used to predict the severity of hepatitis C virus (HCV) infection and characterize the degree of liver fibrosis. In the present study, *MASP1* was upregulated in PSS, and *MASP1* overexpression could inhibit ASFV replication; these results suggest that *MASP1* acts as a host factor restricting ASFV replication and inhibit ASFV replication synergistically with other proteins. *BST2*, being an antiviral protein, acts as a host restriction factor for the broad-spectrum inhibition of enveloped virus replication. *BST2* and actin cytoskeletons are reportedly linked in polarized cells, which can interact with the host reticulin to complete endocytosis [81]. *BST2* can tightly bind HIV particles released from the cell membrane; the retained virus particles are endocytosed and degraded via the interaction between *BCA2/Rabring 7* and *BST2* [82]. These findings from previous studies indicated that *BST2* might induce immune responses and play an important role in resisting viral infection. In the present study, *BST2* was downregulated; its overexpression inhibited ASFV replication. ASFV is an enveloped virus, and *BST2* may play an inhibitory role in the release of its mature virus particles, thereby acting as a host factor restricting ASFV release and subsequently inhibiting its replication.

These results indicated that the components in PSS were involved in the regulation of ASFV virus replication, which was very complicated, and may require the coordination of multiple components, and its specific mechanism was needed further study. This study would provide basic data and biological materials for revealing the mechanism of PSS involved in the regulation of ASFV replication.

## Conclusions

To the best of our knowledge, the present study was the first to demonstrate that PSS could promote ASFV replication. A total of 342 DEPs were screened out from PSS comped with NPSS using iTRAQ technology, PPI networks were constructed, and the potential functions of the identified host proteins were predicted using GO functional annotation and KEGG enrichment analysis. The accuracy of the selected differential proteins was verified at transcript and protein levels, thus providing a basis for subsequent studies. *PCNA* overexpression facilitated viral replication, whereas *MASP1* and *BST2* overexpression inhibited it. This research provided new insights for future detailed research on the pathogenic mechanism and host interactions of ASFV as well as for the development of drugs against ASFV.

## Supplementary Information

**Additional file 1**. Differentially expressed proteins in the serum of pregnant and non-pregnant swine.

## Data Availability

All data generated or analyzed during this study are included in this submitted manuscript.
